# Metabolomic Signature of Coronary Artery Disease in Type 2 Diabetes Mellitus

**DOI:** 10.1155/2017/7938216

**Published:** 2017-03-02

**Authors:** Bernd Stratmann, Katrin Richter, Ruichao Wang, Zhonghao Yu, Tao Xu, Cornelia Prehn, Jerzy Adamski, Thomas Illig, Diethelm Tschoepe, Rui Wang-Sattler

**Affiliations:** ^1^Herz- und Diabeteszentrum NRW, Ruhr-Universitaet Bochum, Diabeteszentrum, 32545 Bad Oeynhausen, Germany; ^2^Research Unit of Molecular Epidemiology, Helmholtz Zentrum München, 85764 Neuherberg, Germany; ^3^Institute of Epidemiology II, Helmholtz Zentrum München, 85764 Neuherberg, Germany; ^4^Institute of Experimental Genetics, Genome Analysis Center, Helmholtz Zentrum München, 85764 Neuherberg, Germany; ^5^Institute of Experimental Genetics, Life and Food Science Center Weihenstephan, Technische Universität München, 85354 Freising, Germany; ^6^German Center for Diabetes Research (DZD), 85764 Neuherberg, Germany

## Abstract

Coronary artery disease (CAD) is a common complication of type 2 diabetes mellitus (T2D). This case-control study was done to identify metabolites with different concentrations between T2D patients with and without CAD and to characterise implicated metabolic mechanisms relating to CAD. Fasting serum samples of 57 T2D subjects, 26 with (cases) and 31 without CAD (controls), were targeted for metabolite profiling of 163 metabolites. To assess the association between metabolite levels and CAD, partial least squares (PLS) analysis and multivariate logistic regression analysis with adjustment for CAD risk factors and medications were performed. We observed a separation of cases and controls with two classes of metabolites being significantly associated with CAD, including phosphatidylcholines, and serine. Four metabolites being independent from the common CAD risk factors displaying best separation between cases and controls were further selected. Addition of the metabolite concentrations to risk factor analysis raised the area under the receiver-operating-characteristic curve from 0.72 to 0.88 (*p* = 0.020), providing improved sensitivity and specificity for CAD classification. Serum phospholipid and serine levels independently discriminate T2D patients with and without CAD. Oxidative stress and reduced antioxidative capacity lead to lower metabolite concentrations probably due to changes in membrane composition and accelerated phospholipid degradation.

## 1. Introduction

Type 2 diabetes mellitus (T2DM) is a complex metabolic disorder which is characterised by abnormal hepatic glucose production, insulin resistance, and impaired pancreatic insulin secretion [1, 2]. Chronic hyperglycaemia in T2DM is associated with both microvascular and macrovascular complications [3], and T2DM is a risk factor for coronary macrovascular disease, autonomic dysfunction, heart failure, and coronary microvascular disease [4].

Coronary artery disease (CAD) is the most common macrovascular complication of cardiovascular disease with an estimated prevalence of 6.9% in men and 6% among women [5]. In 2012, an estimated number of 17.5 million people died from CVDs, accounting for 31% of all global deaths. Of these, an estimated 7.4 million were death due to coronary heart disease. Patients with diabetes mellitus experience a two- to fourfold increased risk of developing CAD or peripheral artery disease (PAD) when comparing with nondiabetic controls [6, 7]. Moreover, associated comorbidities of diabetes mellitus (CAD and hypertension) and metabolic disorders (hyperglycaemia, dyslipidaemia) can contribute to the alteration in diastolic and systolic myocardial function [8]. The underlying mechanism(s) behind the more probable development of coronary macrovascular complications (e.g., CAD and PAD) is still not fully understood. There are several recognised risk factors for CAD, many of them being of metabolic nature [9, 10]. These risk factors include mainly age, gender, BMI, HbA1c, diabetes duration, blood pressure, lipids, brain natriuretic peptide (BNP), and albumin. However, the mechanism(s) cannot be fully explained by the interaction of these risk factors [11]. Besides these well-known risk factors, glycerophospholipids and sphingolipids which are mainly associated with lipoprotein particles contribute to atherogenesis and thus account for the elevated risk of CAD [12, 13]. With the complete mechanistic understanding, individuals being at the highest risk of cardiovascular events could be identified and the progression might be prevented [9, 10, 14, 15].

In search for biomarkers or algorithms predicting the risk of developing cardiovascular disease (CVD), new technologies are applied and screening tools including multifaceted parameters are made available [10, 16–19]. Metabolomics is part of the “omics” research primarily related to the high-throughput identification and quantification of endogenous and exogenous small-molecule metabolites (<1.5 kDa) within a biologic system [20]. The analysis of these metabolites in body fluids like serum and plasma can be used as a promising tool in the diagnostic of diseases [15, 21]. Thus, changes in metabolite profiles are potential sources of biomarkers in terms of reporting alterations in the body due to a disease or drug therapy [14, 22, 23]. For instance, several studies investigated metabolite profiles of subjects with CAD and without CAD, reporting significant differences in acylcarnitines and amino acid concentrations between those two groups of patients [9, 10, 17, 24]. However, these studies did not analyse other classes of metabolites, such as phosphatidylcholines and sphingomyelins which play an important role in membrane function [25], activation of enzymes, and cellular signal transduction [26]. There are only a few studies reporting differences in levels of phosphatidylcholines and sphingomyelins [27–30], though not all subjects were T2D patients in those studies.

Moreover, there were several studies giving evidence that metabolites are heritable in mice [31]. Shah and colleagues strengthened this fact by demonstrating the heritability of the metabolite profiles in human families with early onset of CAD [32]. This could give a hint that CAD could be mediated through metabolites to some extent such as acting as regulatory signal in the control of blood pressure. These metabolic “markers” could give information on involved metabolic pathways which are affected by the disease and help to identify individuals at high risk of the disease and help to optimise screening procedures for the disease as well as for the late complications [14].

In this study, we characterised 163 metabolite concentrations (acylcarnitines, amino acids, phosphatidylcholines, and sphingomyelins) of 57 patients with T2D, including 26 cases with complications of CAD. Our goals were twofold. First, we wanted to identify circulating metabolites discriminating individuals with and without CAD. Second, we aimed at characterising potential molecular mechanisms related to CAD.

## 2. Material and Methods

### 2.1. Study Population

The participants of this study were selected from inpatients at the Herz- und Diabeteszentrum Nordrhein-Westfalen (HDZ NRW) in Bad Oeynhausen after written informed consent. The study cohort included 61 patients who were diagnosed with T2DM according to actual guidelines of the American Diabetes Association (ADA) and the European Association for the Study of Diabetes (EASD). All patients were in fasting state at sampling. Four patients had to be excluded as three of them were reclassified as having type 1 diabetes during recruitment and one having diabetes because of total pancreatectomy. Overall, data on 57 patients with T2DM was available for the analysis.

All patients received oral antidiabetic medication which included the use of either DPP-4 inhibitors (sitagliptin, vildagliptin), sulfonylureas (glimepirides, glibenclamide), thiazolidinediones (pioglitazone), biguanides (metformin), GLP-1-analogon (exenatide), fast or long acting insulin, or a combination of them. Insulin was set as leading compound for classification on treatment as oral alone (oral antidiabetic agent intake) or oral + insulin and insulin alone (insulin therapy). As further medication, patients were taking compromised antihypertensives (*β*-blocker, ACE inhibitors, angiotensin II receptor antagonists, and anticoagulants) and lipid-lowering agents (statins, fibrates). A case-control study design was chosen to compare differences in metabolite concentrations and CAD. Thus, the participants were divided into two groups according to having had a CAD in the past or not. Participants qualified for the CAD group if they had the diagnosis of a clinical vascular disease in at least one vessel defined as a history of myocardial infarction, or a history of coronary, carotid, or peripheral artery revascularisation, or a history of myocardial ischaemia by an exercise stress test, or a history of myocardial ischaemia with any cardiac imaging. Standardised examinations and tests were applied to the study participants including clinical biochemistry and detailed investigation of further heart diseases or other complications according to current medical guidelines.

### 2.2. Sampling

Blood was drawn after an overnight fast (at least 8 hours) in the morning. Within 20 min after sampling, blood was centrifuged (3000 ×g, 4°C, 10 min) for serum collection, flash-frozen, and stored at −80°C until analysis.

### 2.3. Targeted Metabolomics Measurements

Metabolite detection and quantification was conducted in the Metabolomic Platform of the Genome Analysis Center, Helmholtz Zentrum München, using flow injection analysis triple quadrupole mass spectrometry (FIA-MS/MS) and the Absolute*IDQ*™ p150 Kit (Biocrates Life Science AG, Innsbruck, Austria). Out of 10 *μ*L serum, 163 metabolites have been quantified, including free carnitine, 40 acylcarnitines, 14 amino acids, hexoses (sum), 15 lysophosphatidylcholines, 77 phosphatidylcholines, and 15 sphingolipids. A detailed description of the assay procedures and nomenclature have been published previously [33, 34].

Each metabolite had to fulfil the following three criteria to assure data quality [35, 36]: (1) average value of the coefficient of variance (CV) for the metabolite in the three quality controls < 25%, (2) 90% of all measured sample concentrations for the metabolite above limit of detection (LOD), and (3) correlation coefficient between two duplicate measurements of the metabolite in 144 reanalysed samples > 0.5. Out of the 163 measured metabolites, 131 finally passed the quality controls which resulted in a dataset including the sum of hexoses (H1), 14 amino acids (AA), 24 acylcarnitines (AC), 13 SMs, 34 diacyl PCs (PC aa), 37 acyl alkyl PCs (PC ae), and 8 lysoPCs.

### 2.4. Statistical Analyses

The statistical analyses were performed using the statistical package R (version 3.3.1, http://www.r-project.org).

Descriptive characteristics are given as mean ± standard deviation (SD) (Table 1). Clinical characteristics of the study participants were tested for significant difference using a nonparametric Mann-Whitney *U* test. A *p* value smaller than 0.05 was considered as statistically significant.

For all analyses, to ensure comparability between different metabolite levels, the concentrations of metabolite were natural-log transformed and standardized (mean = 0 and SD = 1) [37].

Partial least squares discriminant analysis (PLS-DA) was applied to separate T2D patients with different T2D medication (i.e., insulin treatment and oral antidiabetic agents) as well as the status of CAD.

Logistic regression analysis was conducted to assess the differences in metabolite profiles discriminating independently between subjects with CAD and subjects without CAD. For adjustment in differences in baseline characteristics, variables were chosen based on prior considerations of their clinical relevance with respect to the risk of cardiovascular events (CAD or CHD). Each metabolite was assessed individually. To include potential confounders, we adjusted for two sets of covariates: (1) age and sex as the crude model; (2) age, sex, BMI, HbA1c, diabetes duration, triacylglycerols (TG), LDL : HDL cholesterol ratio, systolic blood pressure (SBP), diastolic blood pressure (DBP), albumin concentrations, medication other than antidiabetic agents (i.e., antihypertensives, lipid-lowering agents), and eGFR [38] as full model. Oral antidiabetics and insulin were not included, because both therapeutic regimens are equally distributed throughout the groups. Smoking, alcohol use, and family history of heart disease could not be used as predictor variables due to lack or weakness of self-reported data.

Metabolites were selected by applying logistic regression analysis, random forest, and a stepwise selection of logistic regression methods. First, metabolites were selected if they met the two following criteria: (1) significant in logistic regression for every metabolite with adjustment for all variables and (2) significant for the 30 most important variables in random forest method where all 131 metabolites and the variables served as covariates. Second, the chosen metabolites were again selected with a stepwise selection of logistic regression according to the Akaike information criterion (AIC). The procedure was performed 100 times, and metabolites that were chosen at least 40 times were kept as the final markers.

To assess the logistic models for classification of cases and controls by a different set of markers (clinical markers, metabolite markers, and both kinds of markers), receiver operating characteristic (ROC) analysis with leave one out (LOO) cross-validation was conducted. LOO method ignores one observation while using the model fitted to the remaining observations to compute the predicted probability for the ignored observation. The cross-validation, drawing of ROC curve, and calculation of the area under the curve (AUC) were performed in R using package bootstrap and ROCR [39], respectively. The different model fits were compared by a likelihood ratio test.

## 3. Results

### 3.1. Characteristics of Study Population

The CAD cases and controls were well matched (Table 1). Thus, the characteristics for clinical parameters between CAD cases and controls were similar as they did not show significant differences in diabetes duration, BMI, HbA1c, proinsulin, C-peptide, SBP, TG, HDL cholesterol, albumin, creatine kinase, CRP, and Lp(a) (*p* > 0.05). However, CAD cases were about 15 years older and included fewer females than controls (Table 1). Values for creatinine, urea, and BNP were significantly higher, and levels for DBP, total cholesterol, LDL cholesterol, and glomerular filtration rate (GFR) were significantly lower in CAD patients compared with patients without CAD (*p* < 0.05). However, patients with CAD tended to show a longer duration of type 2 diabetes and a smaller BMI compared to patients without CAD (*p* < 0.10). The use of oral agents was similar in both groups (six patients with CAD versus 11 patients without CAD), and patients in both groups were more often treated with insulin (20 patients with CAD versus 20 patients without CAD). In the group of CAD patients, lipid-lowering agents and antihypertensives were more often prescribed compared to non-CAD patients.

### 3.2. Differences in Metabolite Profiles Revealed by Partial Least Squares Discriminant Analysis

**Figure 1:**
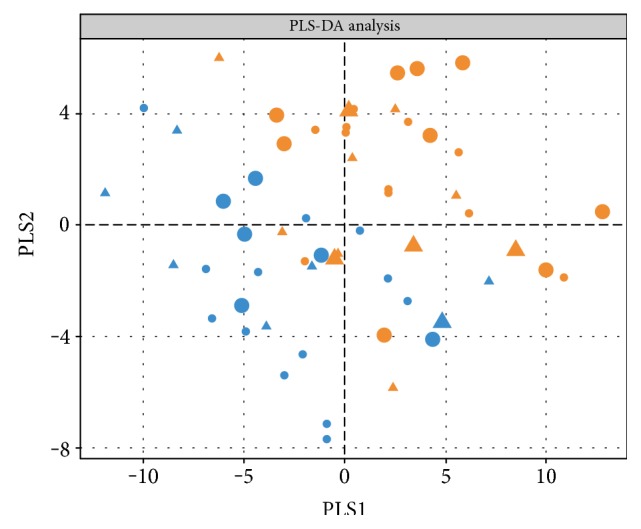
CAD patients separated from non-CAD subjects. PLS-DA (partial least squares discriminant analysis) results are shown. Subjects with CAD are depicted in yellow and subjects without CAD in blue; medication intake is displayed as insulin therapy (circle) or oral antidiabetic agent intake (triangle); sex differences are highlighted in the size of the symbols: females (larger) and males (smaller).

Partial least squares discriminant analysis (PLS-DA) showed a separation of the subjects in two groups (Figure 1): patients with CAD had lower metabolite levels compared to patients without CAD. Considering the sex of the patients, males and females did not show distinct differences in both cases and controls. Moreover, with regard to medication intake, a weak separation could also be observed. In both CAD cases and controls, the metabolite concentrations were lower and accordingly higher in patients treated with insulin compared to patients receiving oral antidiabetic medication.

### 3.3. Identification of Significantly Associated Metabolites with CAD

**Figure 2:**
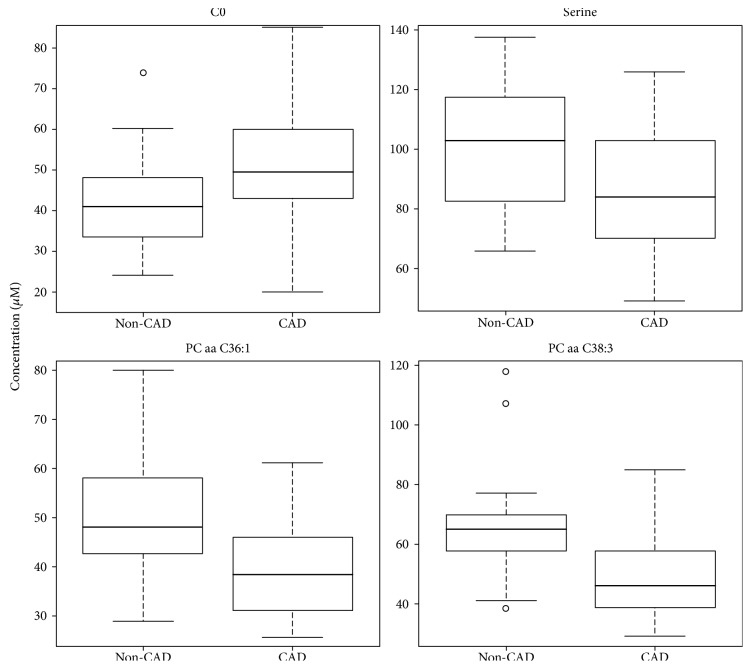
Boxplots of the four identified CAD-specific metabolites. The continuous horizontal line is the median. The lower boundary of the box represents the 25th percentile, and the other boundary represents the 75th percentile. Whiskers above and below the box represent the 95th and 5th percentile.

To further investigate the independent association of metabolites and CAD, multivariate logistic regression analysis was performed for each of the 131 metabolites with adjustment for CAD risk factors, including age, sex, BMI, HbA1c, diabetes duration, TG, LDL : HDL ratio, lipid-lowering agents, SBP, DBP, antihypertensives, albumin, and eGFR. We found 16 metabolites from three different classes of metabolites (1 AA, 11 PCs, and 4 SMs) which significantly differed between subjects with and without CAD (*p* < 0.05) in the crude logistic regression model, for example, PC aa C36:1 (38.95 ± 9.40 *μ*M versus 50.84 ± 12.23 *μ*M, *p* = 0.020) and serine (99.92 ± 20.37 *μ*M versus 86.05 ± 18.89 *μ*M, *p* = 0.045) (see Table S1 in Supplementary Material available online at https://doi.org/10.1155/2017/7938216). For all metabolites, patients with CAD had lower levels compared to those without CAD (Table S1). In the full model, nine metabolites were found significantly associated with CAD (Table S2). Model fit information on coefficients, SEs, and *p* values for the final full model for the 9 significantly related metabolites is provided in Table S3. In addition, we subsequently employed two additional statistical methods, the nonparametric random forest and the parametric stepwise selection, to identify the most significant biomarker candidates. The metabolite selection revealed four metabolites (C0, serine, PC aa C36:1, and PC aa C38:3) showing the most significant (*p* < 0.05) association with CAD (Table 2). For the four selected metabolites, boxplots were drawn to graphically demonstrate the differences in the metabolite concentrations between CAD cases and controls (Figure 2).

### 3.4. Assessment of Model Fit and ROC Curves

**Figure 3:**
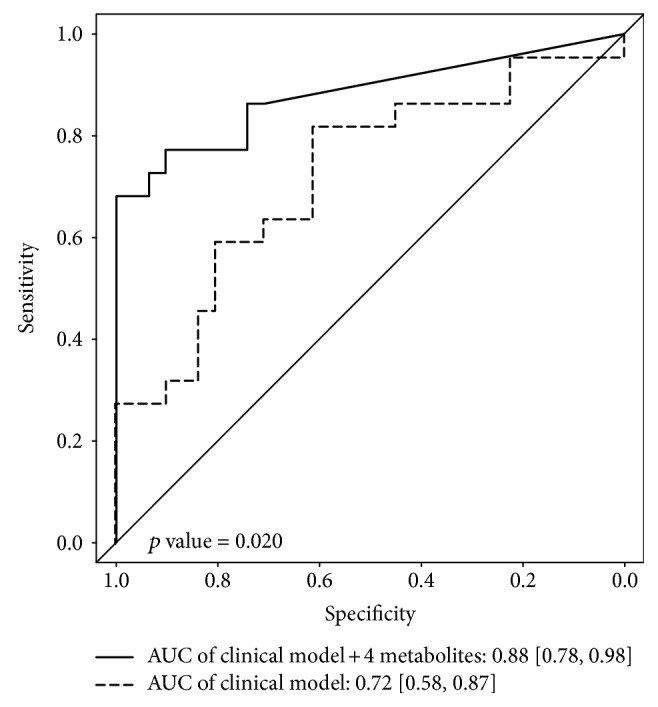
Comparison of sensitivity and specificity with and without the four metabolites. ROC curves and measures of model fit (AUC) are presented for (1) model with clinical parameters (sex, age, BMI, HbA1c, diabetes duration, SBP, DBP, antihypertensives, TG, LDL : HDL ratio, lipid-lowering agents, albumin, and eGFR; dashed line) and (2) model combining the four selected metabolites and the clinical model (solid line).

In order to assess the performance of the chosen model to the data, a ROC curve was calculated (Figure 3). For this, two different models were compared: (1) a model including the clinical covariates only and (2) a model with the combination of the four selected metabolites and the clinical variables. The model with the clinical variables showed a moderate discriminative capability (AUC = 0.72). Both the true and false positive rate improved when adding the four metabolites to the clinical model (AUC = 0.88), confirming the good performance of the chosen model to the data (*p* = 0.020).

## 4. Discussion

We examined the metabolic profiles of type 2 diabetic patients either having a history of CAD or not. Although the number of 57 subjects was rather small, we could demonstrate that serum metabolic profiles were independently associated with CAD after adjustment for multiple clinical covariates. Würtz et al. published results of an NMR metabolomics approach on three population-based cohorts (The National FINRISK Study, Southall and Brent Revisited (SABRE) Study, and British Women's Heart and Health Study) revealing elevated concentrations of phenylalanine and monounsaturated fatty acids and lower concentrations of omega-6 fatty acids and docosahexaenoic acids as being associated with a higher future risk of a cardiovascular event [10]. Our approach was not by NMR but by flow injection analysis mass spectrometry; thus, we identified rather small components in contrast to metabolic intermediates. Recently, Sutter et al. analysed the lipidome of CAD patients in comparison to acute MI patients and healthy controls and identified a series of glycerophospholipids and sphingolipids as being lower in the CAD and MI group compared to healthy controls. Furthermore, they were able to prove that, within a group, statin therapy does not influence the level of the glycerophospholipid and sphingolipid profile [30]. Nevertheless, we included HDL : LDL ratio and presence of lipid-lowering substances of any kind to our regression model. By using plasma lipidomics, Meikle et al. found reduced plasmalogen levels in patients with stable and unstable CAD including a 30% of diabetics patients in the CAD groups, confirming the concept of these metabolites being involved in the clinical setting of CAD [40].

We found nine metabolites being significantly different in type 2 diabetic patients with CAD compared to those without CAD after adjustment for multiple clinical covariates, comprising free carnitine, seven PCs, and serine. Subsequent metabolite selection using random forest and stepwise selection analysis revealed four metabolites (C0, serine, PC aa C36:1, and PC aa C38:3) being the most significantly different between cases and controls. Optimal composition and content of both classes of phospholipids, including PCs, phosphatidylethanolamines (PEs), lysoPCs, and PE-based plasmalogens, are of tremendous importance for maintaining cellular integrity and optimal membrane function [25]. They act in the activation of enzymes, and they are involved in biological signal transduction across the membrane [41]. To this regard, metabolic products of those phospholipids may serve as second messenger in the regulation of cellular function [26]. In addition, phospholipids are highly relevant in key processes such as cell survival, inflammation, and oxygen stress, which are key drivers of vascular diseases [42, 43]. Variations in the content and composition of phospholipids in the membrane are proposed to be associated with cardiac disorders and heart failure as they are related to membrane damage [44]. Phospholipids are metabolised in the heart, and their metabolisation is higher in T2D. Thus, CAD even lowers the free PC content.

There is evidence that changes in circulating phospholipid levels might be associated with the pathology of T2D, dyslipidaemia, and cardiovascular disease [30, 45, 46]. For this reason, changes in serum phospholipid levels may be linked to cardiac dysfunction resembling alterations in their biosynthesis and degradation due to membrane defects.

### 4.1. Phosphatidylcholines

Phosphatidylcholines are the determinant phospholipids in the mammalian heart. They are synthetised via the cytidine diphosphate (CDP) pathway which requires choline and consists of three enzymatic steps [47]. The phosphatidylcholine biosynthesis is regulated by the uptake of choline, energy status of the target tissue, and the modulation of rate-limiting enzymes [48]. Our study showed that several phosphatidylcholines were lower in type 2 diabetic subjects with CAD. These findings suggest that the myocardial membrane was damaged resulting in alterations in phospholipid content and composition. To compensate this defect, phosphatidylcholines are uptaken from circulation in an increased way and serum phosphatidylcholine levels get lower. This is in accordance with the results of Lin and colleagues who reported lower circulation levels of PC 16:0 and PC 18:2 in patients with silent myocardial ischaemia as a consequence of coronary heart disease closely linked to CAD. They justified the lower phosphatidylcholine levels as a result of insufficient supply of ATP and CTP which is coherent with the reduced production of energy in T2D. Furthermore, they observed changes in the membrane, concluding that these alterations originated from ischaemia which caused changes in myocardial enzymes leading to cell membrane damage [28]. Bodi et al. also reported a decrease in fatty acids after ischaemia and a trend towards a downregulated extraction of fatty acids immediately after ischaemia [49]. Low levels of PC plasmalogens have been shown to be associated with oxidative stress, which is common in diabetes and pronounced in diabetes plus CAD [50]. Moreover, significant reductions in choline-containing compounds suggesting a kind of “ischaemic memory” were detected [49]. This might explain the lower levels of phosphatidylcholines in our CAD group.

#### 4.1.1. Serine

Although being a nonessential amino acid, serine is of importance in various biosynthesis pathways like glycine, cysteine, and tryptophan generation, as well as phosphoglyceride-synthesis. Serine is essential in the delivery of C1 fragments in the tetrahydrofolate metabolism. Serine is involved in purine and pyrimidine metabolism as well as in immune reactivity in terms of production of immunoglobulins and antibody synthesis. Besides these functions, serine and threonine are hot spots for O-linked *β*-N-acetylglucosamination [51]. Endo et al. were able to show that increased oxidative stress is associated with increased levels of serine in a mouse model being tolerant to increased oxidative stress by manipulating aldehyde dehydrogenase 2 function. Increased serine concentrations are thus in close relation to protect from increased oxidative stress, which in turn is enhanced by high glucose concentrations [52]. Whether the decreased serine concentrations detected in our CAD cohort are in relationship to a decreased reserve of antioxidative capacity or decreased phospholipid synthesis needs to be evaluated in future studies.

### 4.2. Strengths and Limitations

The limitation of the present study is limited sample size; thus it is not possible to adequately confirm and retrospectively explore subgroups; the study is set up as an explorative approach to identify metabolites being associated with CAD in diabetes. Further investigation in larger samples or cohorts—even prospectively—will provide the detection of more subtle metabolic changes and sufficient precision in the estimates of the utility of each marker to allow for appropriate relative weighting of each component. Furthermore, we did not include patients with acute coronary syndrome. Because of the long recovering period our patients experienced, changes in other metabolite profiles, such as in levels of acylcarnitines or branched chain amino acids as recently associated with CAD by Shah and colleagues, could be neutralised [9].

The strengths of our study are as follows: (1) we have taken great care to evaluate clinical CAD confounders by using a robust clinical model with adjustment for lipids, medication, and kidney function amongst others. (2) We used a high-throughput molecular technology, allowing the detection of various different metabolites. Despite that, the quantification of the serum concentrations of these metabolites needs specialised centres and many laboratories might not have these capabilities [24]. Apart from that, the metabolite profiles show an interindividual and biological variability by race and other demographics [11], for example. Although investigations of determinants of this variability are ongoing, multiple measurements over time may be needed to improve the accuracy of the exact measure of the metabolite concentrations. In addition, sampling specific tissues would be an advantage as they serve as proximal sources of metabolites, enables the localisation of metabolic changes, and may help to gauge the sensitivity and specificity of the signature of metabolic profiles in serum. However, this would be difficult to conduct as this is an invasive procedure.

## 5. Concluding Remarks

In summary, our findings provided evidence that serum phospholipid levels as well as serine levels independently changed in the presence of CAD. Oxidative stress, which is increased in T2D, leads to profound changes in the content and composition of biological membranes and accelerated phospholipid degradation, resulting in lower metabolite levels of PCs and serine. These findings may help to better understand underlying mechanisms of disease, improve stratification of patients at high risk, and optimise screening and diagnosis for the disease and complications. However, this work has to be considered as a pilot for future studies. Accordingly, the performance of more and larger scale metabolomics studies in a prospective setting will help to confirm our findings and to identify new biomarkers for cardiovascular disease.

## Supplementary Material

Table S1 Mean values ± SD for metabolite concentrations (stratified for CAD cases (CAD) and controls (non-CAD)) and ORs ratios for all metabolites being significantly associated with CAD (p<0.05) using logistic regression analysis (with age and gender used as covariates). Table S2 ORs ratios for metabolites being significantly associated with CAD (p<0.05) using logistic regression analysis stratified for CAD class ~ metabolite + age + sex + BMI + HbA1c + Diabetes duration + triglycerides + LDL/HDL-ratio + Albumin + antihypertensive therapy + SBP + DBP + lipid lowering agents + eGFR. Table S3 Coefficients, Standard errors (SEs) and p-values for metabolites being significantly associated with CAD (p<0.05) using logistic regression analysis stratified for CAD class ~ metabolite + age + sex + BMI + HbA1c + Diabetes duration + triglycerides + LDL/HDL-ratio + Albumin + antihypertensive therapy + SBP + DBP + lipid lowering agents + eGFR.

## Figures and Tables

**Table 1:** Characteristics in patients with T2D under antidiabetic therapy (stratified by CAD status; CAD = cases, non-CAD = controls).

Characteristic	CAD *n* = 26	Non-CAD *n* = 31	*p* value^a^
Age (years)	70.0 ± 9.9	55.1 ± 13.2	**1.01E-04**
Males/females (*n*/*n*)	20/6	18/13	
BMI (kg/m^2^)	31.4 ± 4.3	36.3 ± 8.7	0.054
Diabetes duration (years)	18.0 ± 12.7	11.5 ± 7.6	0.065
HbA1c (%)	7.81 ± 1.20	8.85 ± 1.80	0.156
Proinsulin (pmol/l)	14.7 ± 19.2	13.3 ± 7.4	0.146
C-peptide (pmol/l)	1519.2 ± 1202.9	1473.5 ± 939.0	0.654
SBP (mmHg)	129.5 ± 13.2	129.4 ± 13.8	0.949
DBP (mmHg)	70.5 ± 12.6	77.4 ± 10.4	**0.013**
TG (mg/dl)	215.2 ± 156.3	205.8 ± 141.2	0.423
Total cholesterol (mg/dl)	167.5 ± 39.4	189.1 ± 35.5	**5.84E-03**
HDL cholesterol (mg/dl)	38.6 ± 8.9	45.7 ± 24.8	0.251
LDL cholesterol (mg/dl)	95.1 ± 27.3	111.3 ± 35.7	**0.018**
Albumin (mg/l)	16.4 ± 36.6	25.9 ± 39.1	0.082
Creatinine (mg/dl)	1.33 ± 0.52	1.07 ± 0.50	**0.012**
Urea (mg/dl)	61.6 ± 42.5	42.9 ± 18.8	0.037
GFR (ml/min)	60.4 ± 23.2	79.8 ± 29.5	**9.21E-03**
Creatine kinase (U/l)	128.8 ± 79.3	174.4 ± 172.7	0.642
CRP (mg/dl)	1.51 ± 2.66	2.12 ± 4.84	0.438
Lp(a) (mg/dl)	22.8 ± 31.8	40.0 ± 51.7	0.494
BNP (pg/ml)	136.5 ± 178.5	30.9 ± 30.6	**4.68E-06**

Data shown as mean ± SD.

^a^ *p* value for comparing patients with CAD and without CAD using Mann-Whitney *U* test.

**Table 2:** ORs for the selected metabolites using logistic regression analysis in 57 T2D patients. Independent variables used in multiple logistic regression analysis: sex, age, BMI, HbA1c, diabetes duration, SBP, DBP, antihypertensive, triacylglycerols, LDL : HDL ratio, lipid-lowering agents, albumin, and eGFR. ORs are presented as change in concentrations per one SD.

Metabolite	CAD Mean ± SD	Non-CAD Mean ± SD	OR (95% CI)	*p* value
C0	52.17 ± 15.94	42.46 ± 11.72	6.97 [1.70; 61.93]	0.026
Serine	86.05 ± 18.89	99.92 ± 20.37	0.15 [0.01; 0.70]	0.045
PC aa C36:1	38.95 ± 9.40	50.84 ± 12.23	0.16 [0.02; 0.60]	0.020
PC aa C38:3	48.11 ± 13.40	64.26 ± 16.64	0.21 [0.04; 0.81]	0.045
