# Supplementary material for: Metabolomic Signature of Coronary Artery Disease in Type 2 Diabetes Mellitus
Source: Int J Endocrinol. 2017 Mar 2;2017:7938216. doi: 10.1155/2017/7938216 (PMC5350534; doi:10.1155/2017/7938216)
Supplement: Supplementary file 1 — Table S1 Mean values ± SD for metabolite concentrations (stratified for CAD cases (CAD) and controls (non-CAD)) and ORs ratios for all metabolites being significantly associated with CAD (p<0.05) using logistic regression analysis (with age and gender used as covariates). Table S2 ORs ratios for metabolites being significantly associated with CAD (p<0.05) using logistic regression analysis stratified for CAD class ~ metabolite + age + sex + BMI + HbA1c + Diabetes duration + triglycerides + LDL/HDL-ratio + Albumin + antihypertensive therapy + SBP + DBP + lipid lowering agents + eGFR. Table S3 Coefficients, Standard errors (SEs) and p-values for metabolites being significantly associated with CAD (p<0.05) using logistic regression analysis stratified for CAD class ~ metabolite + age + sex + BMI + HbA1c + Diabetes duration + triglycerides + LDL/HDL-ratio + Albumin + antihypertensive therapy + SBP + DBP + lipid lowering agents + eGFR. [file 7938216.f1.docx]

**Table S1** Mean values ± SD for metabolite concentrations (stratified for CAD cases (CAD) and controls (non-CAD)) and ORs ratios for all metabolites being significantly associated with CAD (*p*<0.05) using logistic regression analysis (with age and gender used as covariates)

| **Metabolite** | **CAD**  **Mean ± SD** | **non-CAD**  **Mean ± SD** | **OR (95%CI)** | **p-value** |
| --- | --- | --- | --- | --- |
| Serine | 86.05 ± 18.89 | 99.92 ± 20.37 | 0.42 [0.18;0.88] | 0.031 |
| PC aa C28:1 | 3.023 ± 0.740 | 3.704 ± 0.983 | 0.41 [0.17;0.86] | 0.029 |
| PC aa C36:1 | 38.95 ± 9.40 | 50.84 ± 12.23 | 0.37 [0.15;0.78] | 0.016 |
| PC aa C38:3 | 48.11 ± 13.40 | 64.26 ± 16.64 | 0.36 [0.14;0.79] | 0.017 |
| PC ae C30:2 | 0.144 ± 0.026 | 0.162 ± 0.036 | 0.42 [0.17;0.88] | 0.036 |
| PC ae C34:0 | 1.145 ± 0.314 | 1.378 ± 0.305 | 0.49 [0.23;0.96] | 0.050 |
| PC ae C34:1 | 8.046 ± 1.509 | 9.525 ± 1.984 | 0.42 [0.18;0.86] | 0.025 |
| PC ae C36:1 | 7.225 ± 1.452 | 8.345 ± 1.680 | 0.45 [0.21;0.87] | 0.026 |
| PC ae C38:2 | 1.428 ± 0.283 | 1.701 ± 0.413 | 0.40 [0.17;0.82] | 0.020 |
| PC ae C38:3 | 3.442 ± 0.790 | 4.239 ± 1.048 | 0.47 [0.20;0.94] | 0.048 |
| PC ae C40:2 | 2.002 ± 0.444 | 2.219 ± 0.513 | 0.41 [0.17;0.82] | 0.022 |
| PC ae C40:3 | 0.926 ± 0.184 | 1.065 ± 0.213 | 0.44 [0.19;0.87] | 0.030 |
| SM (OH) C14:1 | 6.741 ± 1.431 | 7.843 ± 1.749 | 0.35 [0.14;0.73] | 0.009 |
| SM (OH) C16:1 | 3.649 ± 0.690 | 4.132 ± 1.020 | 0.38 [0.15;0.82] | 0.021 |
| SM (OH) C22:1 | 14.16 ± 2.55 | 17.20 ± 4.00 | 0.41 [0.16;0.89] | 0.037 |
| SM (OH) C22:2 | 11.49 ± 2.09 | 13.07 ± 2.78 | 0.33 [0.12;0.75] | 0.017 |
|  |  |  |  |  |

**Table S2** ORs ratios for metabolites being significantly associated with CAD (*p*<0.05) using logistic regression analysis stratified for CAD class ~ metabolite + age + sex + BMI + HbA1c + Diabetes duration + triglycerides + LDL/HDL-ratio + Albumin + antihypertensive therapy + SBP + DBP + lipid lowering agents + eGFR

| **Metabolites** | **CAD**  **Mean ± SD** | **Non-CAD**  **Mean ± SD** | **OR (95% CI)** | **p-value** |
| --- | --- | --- | --- | --- |
| C0 | 52.17 ± 15.94 | 42.46 ± 11.72 | 6.97 [1.70; 61.93] | 0.026 |
| Serine | 86.05 ± 18.89 | 99.92 ± 20.37 | 0.15 [0.01;0.70] | 0.045 |
| PC aa C36:1 | 38.95 ± 9.40 | 50.84 ± 12.23 | 0.16 [0.02;0.60] | 0.020 |
| PC aa C38:3 | 48.11 ± 13.40 | 64.26 ± 16.64 | 0.21 [0.04;0.81] | 0.045 |
| PC aa C40:4 | 3.09 ± 0.94 | 4.25 ± 1.33 | 0.17 [0.02;0.69] | 0.036 |
| PC aa C40:5 | 10.35 ± 2.89 | 13.37 ± 3.96 | 0.19 [0.03;0.72] | 0.028 |
| PC aa C42:6 | 0.51 ± 0.12 | 0.57 ± 0.11 | 0.19 [0.02;0.69] | 0.045 |
| PC ae C38:2 | 1.43 ± 0.28 | 1.70 ± 0.41 | 0.18 [0.02;0.66] | 0.033 |
| PC ae C42:2 | 2.00 ± 0.44 | 2.23 ± 0.53 | 0.26 [0.05;0.81] | 0.046 |

**Table S3** Coefficients, Standard errors (SEs) and p-values for metabolites being significantly associated with CAD (*p*<0.05) using logistic regression analysis stratified for CAD class ~ metabolite + age + sex + BMI + HbA1c + Diabetes duration + triglycerides + LDL/HDL-ratio + Albumin + antihypertensive therapy + SBP + DBP + lipid lowering agents + eGFR.

| **Metabolites** | **Coefficients** | **SEs** | **p-value** |
| --- | --- | --- | --- |
| C0 | 1.94 | 0.87 | 0.026 |
| Serine | -1.87 | 0.93 | 0.045 |
| PC aa C36:1 | -1.83 | 0.79 | 0.020 |
| PC aa C38:3 | -1.55 | 0.77 | 0.045 |
| PC aa C40:4 | -1.80 | 0.85 | 0.036 |
| PC aa C40:5 | -1.64 | 0.75 | 0.028 |
| PC aa C42:6 | -1.64 | 0.82 | 0.045 |
| PC ae C38:2 | -1.69 | 0.79 | 0.033 |
| PC ae C42:2 | -1.34 | 0.67 | 0.046 |
