# Supplementary material for: Metabolomic Signature of Coronary Artery Disease in Type 2 Diabetes Mellitus
Source: Int J Endocrinol. 2017 Mar 2;2017:7938216. doi: 10.1155/2017/7938216 (PMC5350534; doi:10.1155/2017/7938216)
Supplement: Supplementary file 2 [file 7938216.f2.pdf]

Table S1 Mean values ± SD for metabolite concentrations (stratified for CAD cases (CAD) and controls (non-CAD)) and ORs ratios for all metabolites being significantly associated with CAD (p<0.05) using logistic regression analysis (with age and gender used as covariates)

**Metabolite**	**CAD Mean ± SD**	**non-CAD Mean ± SD**	**OR (95%CI)**	**p-value**
Serine	86.05 ± 18.89	99.92 ± 20.37	0.42 [0.18;0.88]	0.031
PC aa C28:1	3.023 ± 0.740	3.704 ± 0.983	0.41 [0.17;0.86]	0.029
PC aa C36:1	38.95 ± 9.40	50.84 ± 12.23	0.37 [0.15;0.78]	0.016
PC aa C38:3	48.11 ± 13.40	64.26 ± 16.64	0.36 [0.14;0.79]	0.017
PC ae C30:2	0.144 ± 0.026	0.162 ± 0.036	0.42 [0.17;0.88]	0.036
PC ae C34:0	1.145 ± 0.314	1.378 ± 0.305	0.49 [0.23;0.96]	0.050
PC ae C34:1	8.046 ± 1.509	9.525 ± 1.984	0.42 [0.18;0.86]	0.025
PC ae C36:1	7.225 ± 1.452	8.345 ± 1.680	0.45 [0.21;0.87]	0.026
PC ae C38:2	1.428 ± 0.283	1.701 ± 0.413	0.40 [0.17;0.82]	0.020
PC ae C38:3	3.442 ± 0.790	4.239 ± 1.048	0.47 [0.20;0.94]	0.048
PC ae C40:2	2.002 ± 0.444	2.219 ± 0.513	0.41 [0.17;0.82]	0.022
PC ae C40:3	0.926 ± 0.184	1.065 ± 0.213	0.44 [0.19;0.87]	0.030
SM (OH) C14:1	6.741 ± 1.431	7.843 ± 1.749	0.35 [0.14;0.73]	0.009
SM (OH) C16:1	3.649 ± 0.690	4.132 ± 1.020	0.38 [0.15;0.82]	0.021
SM (OH) C22:1	14.16 ± 2.55	17.20 ± 4.00	0.41 [0.16;0.89]	0.037
SM (OH) C22:2	11.49 ± 2.09	13.07 ± 2.78	0.33 [0.12;0.75]	0.017

Table S2 ORs ratios for metabolites being significantly associated with CAD (p<0.05) using logistic regression analysis stratified for CAD class ~ metabolite + age + sex + BMI + HbA1c + Diabetes duration + triglycerides + LDL/HDL-ratio + Albumin + antihypertensive therapy + SBP + DBP + lipid lowering agents + eGFR

**Metabolites**	**CAD Mean ± SD**	**Non-CAD Mean ± SD**	**OR (95% CI)**	**p-value**
C0	52.17 ± 15.94	42.46 ± 11.72	6.97 [1.70; 61.93]	0.026
Serine	86.05 ± 18.89	99.92 ± 20.37	0.15 [0.01;0.70]	0.045
PC aa C36:1	38.95 ± 9.40	50.84 ± 12.23	0.16 [0.02;0.60]	0.020
PC aa C38:3	48.11 ± 13.40	64.26 ± 16.64	0.21 [0.04;0.81]	0.045
PC aa C40:4	3.09 ± 0.94	4.25 ± 1.33	0.17 [0.02;0.69]	0.036
PC aa C40:5	10.35 ± 2.89	13.37 ± 3.96	0.19 [0.03;0.72]	0.028
PC aa C42:6	0.51 ± 0.12	0.57 ± 0.11	0.19 [0.02;0.69]	0.045
PC ae C38:2	1.43 ± 0.28	1.70 ± 0.41	0.18 [0.02;0.66]	0.033
PC ae C42:2	2.00 ± 0.44	2.23 ± 0.53	0.26 [0.05;0.81]	0.046

Table S3 Coefficients, Standard errors (SEs) and p-values for metabolites being significantly associated with CAD (p<0.05) using logistic regression analysis stratified for CAD class ~ metabolite + age + sex + BMI + HbA1c + Diabetes duration + triglycerides + LDL/HDL-ratio + Albumin + antihypertensive therapy + SBP + DBP + lipid lowering agents + eGFR.

Metabolites	Coefficients	SEs	p-value
C0	1.94	0.87	0.026
Serine	-1.87	0.93	0.045
PC aa C36:1	-1.83	0.79	0.020
PC aa C38:3	-1.55	0.77	0.045
PC aa C40:4	-1.80	0.85	0.036
PC aa C40:5	-1.64	0.75	0.028
PC aa C42:6	-1.64	0.82	0.045
PC ae C38:2	-1.69	0.79	0.033
PC ae C42:2	-1.34	0.67	0.046
